# Diaqua­bis(*N*,*N*-diethyl­nicotinamide-κ*N* ^1^)bis­(4-formyl­benzoato-κ*O*)nickel(II)

**DOI:** 10.1107/S1600536809006345

**Published:** 2009-02-25

**Authors:** Mustafa Sertçelik, Barış Tercan, Ertan Şahin, Hacali Necefoğlu, Tuncer Hökelek

**Affiliations:** a Kafkas University, Department of Chemistry, 63100 Kars, Turkey; b Karabük University, Department of Physics, 78050 Karabük, Turkey; c Atatürk University, Department of Chemistry, 22240 Erzurum, Turkey; d Hacettepe University, Department of Physics, 06800 Beytepe, Ankara, Turkey

## Abstract

In the title centrosymmetric mononuclear Ni^II^ compound, [Ni(C_8_H_5_O_3_)_2_(C_10_H_14_N_2_O)_2_(H_2_O)_2_], the central Ni^II^ atom is coordinated by two O atoms from two 4-formyl­benzoate (FOB) ligands, two O atoms from two water mol­ecules and two N atoms from two diethyl­nicotinamide (DENA) ligands. The coordination geometry is slightly distorted octa­hedral, with four O atoms in the equatorial plane and two N atoms in axial positions. Intra­molecular O—H⋯O hydrogen bonds are observed. In the crystal structure, mol­ecules are linked into chains along the *a* axis by inter­molecular O—H⋯O hydrogen bonds. The structure is further stabilized by π–π inter­actions between the pyridine rings of DENA units, with a centroid–centroid distance of 3.668 (2) Å.

## Related literature

For general background, see: Antolini *et al.* (1982 ▶); Bigoli *et al.* (1972 ▶); Nadzhafov *et al.* (1981 ▶); Shnulin *et al.* (1981 ▶). For related structures, see: Hökelek *et al.* (1995 ▶, 1997 ▶, 2007 ▶, 2008 ▶); Hökelek & Necefouglu (1996 ▶, 1997 ▶); Hökelek & Necefoğlu (2007 ▶); Sertçelik *et al.* (2009 ▶).
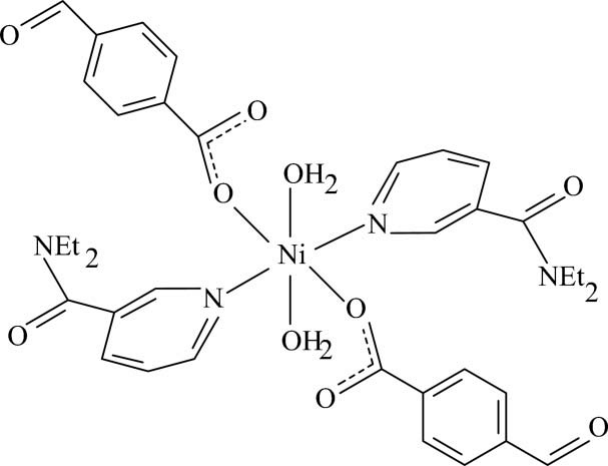


## Experimental

### 

#### Crystal data

[Ni(C_8_H_5_O_3_)_2_(C_10_H_14_N_2_O)_2_(H_2_O)_2_]
*M* *_r_* = 749.43
Triclinic, 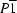

*a* = 7.2909 (2) Å
*b* = 8.6883 (3) Å
*c* = 15.9037 (4) Å
α = 85.034 (5)°
β = 78.576 (4)°
γ = 67.594 (3)°
*V* = 912.85 (5) Å^3^
*Z* = 1
Mo *K*α radiation
μ = 0.59 mm^−1^
*T* = 294 K
0.35 × 0.20 × 0.15 mm

#### Data collection

Rigaku R-AXIS RAPID-S diffractometer
Absorption correction: multi-scan (Blessing, 1995 ▶) *T*_min_ = 0.870, *T*_max_ = 0.918
19676 measured reflections
3740 independent reflections
2797 reflections with *I* > 2σ(*I*)
*R*_int_ = 0.098

#### Refinement

*R*[*F* ^2^ > 2σ(*F* ^2^)] = 0.062
*wR*(*F* ^2^) = 0.122
*S* = 1.04
3740 reflections
242 parameters
3 restraints
H atoms treated by a mixture of independent and constrained refinement
Δρ_max_ = 0.50 e Å^−3^
Δρ_min_ = −0.31 e Å^−3^

### 

Data collection: *CrystalClear* (Rigaku/MSC, 2005 ▶); cell refinement: *CrystalClear*; data reduction: *CrystalClear*; program(s) used to solve structure: *SHELXS97* (Sheldrick, 2008 ▶); program(s) used to refine structure: *SHELXL97* (Sheldrick, 2008 ▶); molecular graphics: *ORTEP-3 for Windows* (Farrugia, 1997 ▶) and *Mercury* (Macrae *et al.*, 2006 ▶); software used to prepare material for publication: *WinGX* (Farrugia, 1999 ▶).

## Supplementary Material

Crystal structure: contains datablocks I, global. DOI: 10.1107/S1600536809006345/ci2769sup1.cif

Structure factors: contains datablocks I. DOI: 10.1107/S1600536809006345/ci2769Isup2.hkl

Additional supplementary materials:  crystallographic information; 3D view; checkCIF report

## Figures and Tables

**Table 1:** Selected bond lengths (Å)

O5—Ni1	2.084 (2)
Ni1—O1	2.069 (2)
Ni1—N1	2.100 (3)

**Table 2:** Hydrogen-bond geometry (Å, °)

*D*—H⋯*A*	*D*—H	H⋯*A*	*D*⋯*A*	*D*—H⋯*A*
O5—H51⋯O4^i^	0.84 (2)	1.97 (2)	2.796 (4)	170 (3)
O5—H52⋯O2	0.85 (3)	1.81 (3)	2.646 (4)	168 (4)

Symmetry code: (i) 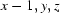.

**Fig. 1.**
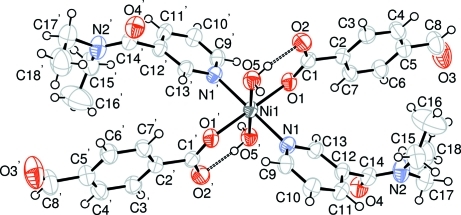
The molecular structure of the title compound with the atom-numbering scheme. Displacement ellipsoids are drawn at the 50% probability level. Hydrogen bonds are shown as dashed lines. Primed atoms are generated by the symmetry operator (-x, -y, -z).

**Fig. 2.**
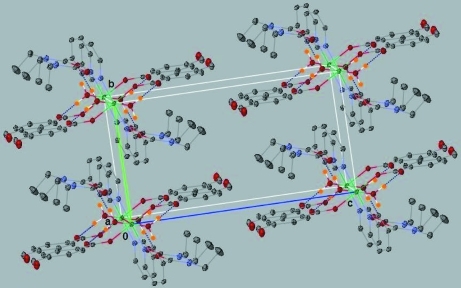
A partial packing diagram of the title compound viewed down the a axis, showing hydrogen bonds (dotted lines) linking the molecules into chains. H atoms not involved in hydrogen bonding have been omitted.

## supplementary crystallographic information

### Comment

The nicotinic acid derivative *N*,*N*-diethylnicotinamide (DENA) is an important respiratory stimulant (Bigoli *et al.*, 1972). The structural functions and coordination relationships of the arylcarboxylate ion in transition metal complexes of benzoic acid derivatives change depending on the nature and position of the substituent groups on the benzene ring, the nature of the additional ligand molecule or solvent, and the medium of the synthesis (Nadzhafov *et al.*, 1981; Shnulin *et al.*, 1981). Transition metal complexes with biochemically active ligands frequently show interesting physical and/or chemical properties, as a result they may find applications in biological systems (Antolini *et al.*, 1982). The structure determination of the title compound, a nickel complex with two formylbenzoate (FOB), two diethylnicotinamide (DENA) ligands and two water molecules, was undertaken in order to determine the properties of the ligands and also to compare the results obtained with those reported previously.

The title compound is a monomeric complex, with the Ni^II^ atom on a centre of symmetry (Fig. 1). All ligands are monodentate. The four O atoms (O1, O5, and the symmetry-related atoms, O1', O5') lie in the equatorial plane around the Ni1 atom forming a slightly distorted square-planar arrangement, while the slightly distorted octahedral coordination is completed by the two N atoms of the DENA ligands (N1, N1') in the axial positions (Table 1 and Fig. 1). An intramolecular O—H···O hydrogen bond (Table 2) results in the formation of a six-membered ring Ni1/O1/O2/O5/C1/H52 ring.

The near equality of the C1—O1 [1.263 (4) Å] and C1—O2 [1.249 (4) Å] bonds in the carboxylate group indicates a delocalized bonding arrangement, rather than localized single and double bonds, and may be compared with the corresponding distances: 1.262 (3) and 1.249 (3) Å in [Mn(DENA)_2_(C_8_H_5_O_3_)_2_(H_2_O)_2_] (Sertçelik *et al.*, 2009), 1.256 (6) and 1.245 (6) Å in [Mn(DENA)_2_(C_7_H_4_ClO_2_)_2_(H_2_O)_2_] (Hökelek *et al.*, 2008), 1.265 (6) and 1.275 (6) Å in [Mn(C_9_H_10_NO_2_)_2_(H_2_O)_4_].2H_2_O (Hökelek & Necefoğlu, 2007), 1.260 (4) and 1.252 (4) Å in [Zn(DENA)_2_(C_7_H_4_FO_2_)_2_(H_2_O)_2_] (Hökelek *et al.*, 2007), 1.259 (9) and 1.273 (9) Å in Cu_2_(DENA)_2_(C_6_H_5_COO)_4_ (Hökelek *et al.*, 1995), 1.279 (4) and 1.246 (4) Å in [Zn_2_(DENA)_2_(C_7_H_5_O_3_)_4_].2H_2_O (Hökelek & Necefoğlu, 1996), 1.251 (6) and 1.254 (7) Å in [Co(DENA)_2_(C_7_H_5_O_3_)_2_(H_2_O)_2_] (Hökelek & Necefoğlu, 1997), 1.278 (3) and 1.246 (3) Å in [Cu(DENA)_2_(C_7_H_4_NO_4_)_2_(H_2_O)_2_] (Hökelek *et al.*, 1997). The average Ni—O bond length in the title complex is 2.077 (3) Å and the Ni1 atom is displaced out of the least-squares plane of the carboxylate group (O1/C1/O2) by 0.732 (1) Å. The dihedral angle between the planar carboxylate group and the C2-C7 benzene ring is 4.3 (3)°.

In the crystal structure, intermolecular O—H···O hydrogen bonds (Table 1) link the molecules into infinite chains (Fig. 2) along the *a* axis, which may be effective in the stabilization of the structure. A π-π contact is also observed between the pyridine rings (N1/C9—C13, centroid *Cg*1) of DENA units, with a *Cg*1···*Cg*1^i^ [symmetry code: (i) 1-*x*, -1-*y*, -*z*] distance of 3.668 (2) Å.

### Experimental

The title compound was prepared by the reaction of Ni(SO_4_)H_2_O (1.73 g, 10 mmol) in H_2_O (50 ml) and DENA (3.56 g, 20 mmol) in H_2_O (15 ml) with sodium 4-formylbenzoate (3.44 g, 20 mmol) in H_2_O (50 ml). The mixture was filtered and set aside to crystallize at ambient temperature for several days, giving green single crystals.

### Refinement

H atoms of water molecule were located in a difference Fourier map and refined isotropically, with O–H and H···H distances restrained to 0.84 (1) Å and 1.37 (2) Å, respectively. The remaining H atoms were positioned geometrically with C—H = 0.93, 0.97 and 0.96 Å, for aromatic, methylene and methyl H atoms and constrained to ride on their parent atoms, with *U*_iso_(H) = *xU*_eq_(C), where *x* = 1.5 for methyl H atoms and *x* = 1.2 for all other H atoms.

### Crystal data

**Table:**

[Ni(C_8_H_5_O_3_)_2_(C_10_H_14_N_2_O)_2_(H_2_O)_2_]	*Z* = 1
*M**_r_* = 749.43	*F*(000) = 394
Triclinic, *P*1	*D*_x_ = 1.363 Mg m^−^^3^
Hall symbol: -P 1	Mo *K*α radiation, λ = 0.71073 Å
*a* = 7.2909 (2) Å	Cell parameters from 4227 reflections
*b* = 8.6883 (3) Å	θ = 2.5–26.4°
*c* = 15.9037 (4) Å	µ = 0.59 mm^−^^1^
α = 85.034 (5)°	*T* = 294 K
β = 78.576 (4)°	Prism, green
γ = 67.594 (3)°	0.35 × 0.20 × 0.15 mm
*V* = 912.85 (5) Å^3^	

### Data collection

**Table:**

Rigaku R-AXIS RAPID-S diffractometer	3740 independent reflections
Radiation source: fine-focus sealed tube	2797 reflections with *I* > 2σ(*I*)
graphite	*R*_int_ = 0.098
ω scans	θ_max_ = 26.4°, θ_min_ = 2.5°
Absorption correction: multi-scan (Blessing, 1995)	*h* = −9→9
*T*_min_ = 0.870, *T*_max_ = 0.918	*k* = −10→10
19676 measured reflections	*l* = −19→19

### Refinement

**Table:**

Refinement on *F*^2^	Primary atom site location: structure-invariant direct methods
Least-squares matrix: full	Secondary atom site location: difference Fourier map
*R*[*F*^2^ > 2σ(*F*^2^)] = 0.062	Hydrogen site location: inferred from neighbouring sites
*wR*(*F*^2^) = 0.122	H atoms treated by a mixture of independent and constrained refinement
*S* = 1.04	*w* = 1/[σ^2^(*F*_o_^2^) + (0.0235*P*)^2^ + 0.8051*P*] where *P* = (*F*_o_^2^ + 2*F*_c_^2^)/3
3740 reflections	(Δ/σ)_max_ = 0.001
242 parameters	Δρ_max_ = 0.50 e Å^−^^3^
3 restraints	Δρ_min_ = −0.31 e Å^−^^3^

### Special details

**Table:**

Geometry. All esds (except the esd in the dihedral angle between two l.s. planes) are estimated using the full covariance matrix. The cell esds are taken into account individually in the estimation of esds in distances, angles and torsion angles; correlations between esds in cell parameters are only used when they are defined by crystal symmetry. An approximate (isotropic) treatment of cell esds is used for estimating esds involving l.s. planes.
Refinement. Refinement of *F*^2^ against ALL reflections. The weighted *R*-factor *wR* and goodness of fit *S* are based on *F*^2^, conventional *R*-factors *R* are based on *F*, with *F* set to zero for negative *F*^2^. The threshold expression of *F*^2^ > σ(*F*^2^) is used only for calculating *R*-factors(gt) *etc*. and is not relevant to the choice of reflections for refinement. *R*-factors based on *F*^2^ are statistically about twice as large as those based on *F*, and *R*- factors based on ALL data will be even larger.

### Fractional atomic coordinates and isotropic or equivalent isotropic displacement parameters (Å^2^)

**Table:**

	*x*	*y*	*z*	*U*_iso_*/*U*_eq_	
O5	−0.2711 (4)	−0.0197 (4)	0.06069 (16)	0.0494 (6)	
H52	−0.284 (6)	0.028 (4)	0.1070 (13)	0.069 (14)*	
H51	−0.260 (6)	−0.1180 (19)	0.074 (2)	0.072 (15)*	
Ni1	0.0000	0.0000	0.0000	0.03948 (19)	
O1	0.0244 (3)	0.1122 (3)	0.10462 (14)	0.0465 (6)	
O2	−0.2567 (4)	0.1272 (3)	0.19683 (16)	0.0569 (7)	
N1	0.1775 (4)	−0.2306 (3)	0.04762 (17)	0.0427 (7)	
O4	0.7320 (4)	−0.3311 (3)	0.12366 (16)	0.0579 (7)	
C1	−0.0812 (5)	0.1254 (4)	0.1788 (2)	0.0430 (8)	
C12	0.4355 (5)	−0.3815 (4)	0.1297 (2)	0.0423 (8)	
C13	0.3191 (5)	−0.2372 (4)	0.0918 (2)	0.0433 (8)	
H13	0.3401	−0.1398	0.0974	0.052*	
C7	0.2092 (5)	0.1420 (4)	0.2370 (2)	0.0467 (8)	
H7	0.2787	0.1384	0.1810	0.056*	
C9	0.1550 (5)	−0.3738 (4)	0.0380 (2)	0.0467 (8)	
H9	0.0593	−0.3722	0.0067	0.056*	
C2	0.0178 (5)	0.1355 (4)	0.2521 (2)	0.0420 (8)	
N2	0.6115 (5)	−0.4182 (4)	0.2506 (2)	0.0617 (9)	
C3	−0.0823 (5)	0.1368 (4)	0.3360 (2)	0.0502 (9)	
H3	−0.2093	0.1304	0.3472	0.060*	
C11	0.4075 (5)	−0.5277 (4)	0.1196 (2)	0.0477 (9)	
H11	0.4824	−0.6269	0.1445	0.057*	
O3	0.4414 (5)	0.1916 (5)	0.4558 (2)	0.0974 (11)	
C14	0.6023 (5)	−0.3740 (4)	0.1690 (2)	0.0474 (9)	
C4	0.0071 (6)	0.1476 (5)	0.4030 (2)	0.0559 (10)	
H4	−0.0606	0.1483	0.4591	0.067*	
C6	0.2976 (5)	0.1538 (5)	0.3041 (2)	0.0523 (9)	
H6	0.4251	0.1592	0.2932	0.063*	
C10	0.2674 (5)	−0.5233 (4)	0.0723 (2)	0.0505 (9)	
H10	0.2483	−0.6202	0.0635	0.061*	
C5	0.1956 (6)	0.1575 (5)	0.3878 (2)	0.0533 (9)	
C17	0.7912 (7)	−0.4225 (6)	0.2841 (3)	0.0746 (13)	
H17A	0.8047	−0.4912	0.3356	0.090*	
H17B	0.9119	−0.4723	0.2417	0.090*	
C15	0.4604 (7)	−0.4666 (6)	0.3106 (3)	0.0740 (13)	
H15A	0.3689	−0.4823	0.2785	0.089*	
H15B	0.5285	−0.5725	0.3373	0.089*	
C16	0.3433 (9)	−0.3450 (9)	0.3777 (4)	0.137 (3)	
H16A	0.2414	−0.3797	0.4119	0.206*	
H16B	0.2804	−0.2383	0.3518	0.206*	
H16C	0.4309	−0.3367	0.4134	0.206*	
C8	0.2863 (7)	0.1717 (6)	0.4606 (3)	0.0767 (13)	
H8	0.2159	0.1645	0.5154	0.092*	
C18	0.7725 (8)	−0.2525 (6)	0.3038 (3)	0.0961 (17)	
H18A	0.8938	−0.2582	0.3212	0.144*	
H18B	0.6600	−0.2068	0.3494	0.144*	
H18C	0.7518	−0.1827	0.2537	0.144*	

### Atomic displacement parameters (Å^2^)

**Table:**

	*U*^11^	*U*^22^	*U*^33^	*U*^12^	*U*^13^	*U*^23^
O5	0.0454 (14)	0.0569 (17)	0.0485 (16)	−0.0216 (13)	−0.0106 (12)	0.0032 (14)
Ni1	0.0359 (3)	0.0445 (4)	0.0398 (4)	−0.0152 (3)	−0.0123 (3)	0.0044 (3)
O1	0.0473 (14)	0.0542 (15)	0.0420 (14)	−0.0207 (12)	−0.0141 (11)	0.0019 (11)
O2	0.0440 (14)	0.0733 (18)	0.0544 (15)	−0.0224 (13)	−0.0070 (12)	−0.0074 (13)
N1	0.0374 (15)	0.0452 (17)	0.0475 (17)	−0.0160 (13)	−0.0122 (12)	0.0030 (13)
O4	0.0500 (15)	0.0703 (18)	0.0611 (16)	−0.0315 (14)	−0.0132 (13)	0.0098 (14)
C1	0.0424 (19)	0.0387 (19)	0.046 (2)	−0.0103 (15)	−0.0139 (16)	0.0022 (15)
C12	0.0368 (18)	0.050 (2)	0.0411 (19)	−0.0174 (16)	−0.0105 (15)	0.0058 (16)
C13	0.0418 (18)	0.0436 (19)	0.047 (2)	−0.0158 (16)	−0.0144 (16)	0.0009 (16)
C7	0.048 (2)	0.053 (2)	0.0386 (19)	−0.0192 (17)	−0.0045 (15)	−0.0002 (16)
C9	0.046 (2)	0.054 (2)	0.046 (2)	−0.0236 (18)	−0.0159 (16)	0.0037 (17)
C2	0.0421 (19)	0.0426 (19)	0.0410 (19)	−0.0144 (16)	−0.0106 (15)	0.0013 (15)
N2	0.061 (2)	0.080 (2)	0.057 (2)	−0.0371 (18)	−0.0232 (16)	0.0143 (18)
C3	0.044 (2)	0.057 (2)	0.048 (2)	−0.0182 (18)	−0.0081 (16)	0.0019 (18)
C11	0.0444 (19)	0.046 (2)	0.051 (2)	−0.0137 (17)	−0.0145 (16)	0.0106 (17)
O3	0.101 (3)	0.155 (3)	0.069 (2)	−0.076 (3)	−0.0352 (19)	0.005 (2)
C14	0.044 (2)	0.050 (2)	0.050 (2)	−0.0176 (17)	−0.0140 (17)	0.0077 (17)
C4	0.062 (2)	0.066 (3)	0.038 (2)	−0.024 (2)	−0.0041 (17)	−0.0028 (18)
C6	0.051 (2)	0.061 (2)	0.051 (2)	−0.0257 (19)	−0.0137 (17)	−0.0005 (18)
C10	0.054 (2)	0.046 (2)	0.057 (2)	−0.0224 (18)	−0.0179 (18)	0.0045 (17)
C5	0.058 (2)	0.062 (2)	0.044 (2)	−0.025 (2)	−0.0139 (18)	0.0018 (18)
C17	0.081 (3)	0.078 (3)	0.076 (3)	−0.033 (3)	−0.038 (2)	0.014 (2)
C15	0.081 (3)	0.092 (3)	0.057 (3)	−0.040 (3)	−0.019 (2)	0.009 (2)
C16	0.102 (5)	0.176 (7)	0.131 (5)	−0.059 (5)	0.026 (4)	−0.069 (5)
C8	0.081 (3)	0.106 (4)	0.054 (3)	−0.042 (3)	−0.024 (2)	−0.003 (2)
C18	0.128 (5)	0.085 (4)	0.094 (4)	−0.046 (3)	−0.049 (3)	−0.002 (3)

### Geometric parameters (Å, °)

**Table:**

O5—Ni1	2.084 (2)	N2—C17	1.496 (5)
O5—H52	0.85 (3)	C3—C4	1.380 (5)
O5—H51	0.84 (2)	C3—H3	0.93
Ni1—O1	2.069 (2)	C11—C10	1.371 (5)
Ni1—O1^i^	2.069 (2)	C11—H11	0.93
Ni1—O5^i^	2.084 (2)	O3—C8	1.194 (5)
Ni1—N1	2.100 (3)	C4—C5	1.382 (5)
Ni1—N1^i^	2.100 (3)	C4—H4	0.93
O1—C1	1.263 (4)	C6—C5	1.386 (5)
O2—C1	1.249 (4)	C6—H6	0.93
N1—C9	1.340 (4)	C10—H10	0.93
N1—C13	1.341 (4)	C5—C8	1.478 (5)
O4—C14	1.227 (4)	C17—C18	1.486 (6)
C1—C2	1.511 (4)	C17—H17A	0.97
C12—C13	1.384 (4)	C17—H17B	0.97
C12—C11	1.389 (5)	C15—C16	1.459 (6)
C12—C14	1.498 (4)	C15—H15A	0.97
C13—H13	0.93	C15—H15B	0.97
C7—C6	1.380 (4)	C16—H16A	0.96
C7—C2	1.391 (4)	C16—H16B	0.96
C7—H7	0.93	C16—H16C	0.96
C9—C10	1.379 (5)	C8—H8	0.93
C9—H9	0.93	C18—H18A	0.96
C2—C3	1.388 (5)	C18—H18B	0.96
N2—C14	1.328 (4)	C18—H18C	0.96
N2—C15	1.473 (5)		
			
Ni1—O5—H52	98 (3)	C10—C11—C12	118.8 (3)
Ni1—O5—H51	113 (3)	C10—C11—H11	120.6
H52—O5—H51	106 (2)	C12—C11—H11	120.6
O1—Ni1—O1^i^	180.00 (6)	O4—C14—N2	121.3 (3)
O1—Ni1—O5	92.91 (10)	O4—C14—C12	118.6 (3)
O1^i^—Ni1—O5	87.09 (10)	N2—C14—C12	120.1 (3)
O1—Ni1—O5^i^	87.09 (10)	C3—C4—C5	121.0 (3)
O1^i^—Ni1—O5^i^	92.91 (10)	C3—C4—H4	119.5
O5—Ni1—O5^i^	180.00 (19)	C5—C4—H4	119.5
O1—Ni1—N1	88.53 (10)	C7—C6—C5	119.8 (3)
O1^i^—Ni1—N1	91.47 (10)	C7—C6—H6	120.1
O5—Ni1—N1	93.76 (10)	C5—C6—H6	120.1
O5^i^—Ni1—N1	86.24 (10)	C11—C10—C9	119.3 (3)
O1—Ni1—N1^i^	91.47 (10)	C11—C10—H10	120.3
O1^i^—Ni1—N1^i^	88.53 (10)	C9—C10—H10	120.3
O5—Ni1—N1^i^	86.24 (10)	C4—C5—C6	119.4 (3)
O5^i^—Ni1—N1^i^	93.76 (10)	C4—C5—C8	119.8 (4)
N1—Ni1—N1^i^	180.0 (2)	C6—C5—C8	120.8 (4)
C1—O1—Ni1	126.8 (2)	C18—C17—N2	111.3 (4)
C9—N1—C13	117.2 (3)	C18—C17—H17A	109.4
C9—N1—Ni1	123.6 (2)	N2—C17—H17A	109.4
C13—N1—Ni1	119.3 (2)	C18—C17—H17B	109.4
O2—C1—O1	125.9 (3)	N2—C17—H17B	109.4
O2—C1—C2	117.7 (3)	H17A—C17—H17B	108.0
O1—C1—C2	116.3 (3)	C16—C15—N2	113.6 (4)
C13—C12—C11	118.2 (3)	C16—C15—H15A	108.8
C13—C12—C14	117.4 (3)	N2—C15—H15A	108.8
C11—C12—C14	123.8 (3)	C16—C15—H15B	108.8
N1—C13—C12	123.4 (3)	N2—C15—H15B	108.8
N1—C13—H13	118.3	H15A—C15—H15B	107.7
C12—C13—H13	118.3	C15—C16—H16A	109.5
C6—C7—C2	120.9 (3)	C15—C16—H16B	109.5
C6—C7—H7	119.6	H16A—C16—H16B	109.5
C2—C7—H7	119.6	C15—C16—H16C	109.5
N1—C9—C10	123.0 (3)	H16A—C16—H16C	109.5
N1—C9—H9	118.5	H16B—C16—H16C	109.5
C10—C9—H9	118.5	O3—C8—C5	126.2 (4)
C3—C2—C7	119.0 (3)	O3—C8—H8	116.9
C3—C2—C1	119.8 (3)	C5—C8—H8	116.9
C7—C2—C1	121.2 (3)	C17—C18—H18A	109.5
C14—N2—C15	125.2 (3)	C17—C18—H18B	109.5
C14—N2—C17	117.6 (3)	H18A—C18—H18B	109.5
C15—N2—C17	117.1 (3)	C17—C18—H18C	109.5
C4—C3—C2	119.9 (3)	H18A—C18—H18C	109.5
C4—C3—H3	120.1	H18B—C18—H18C	109.5
C2—C3—H3	120.1		
			
O5—Ni1—O1—C1	10.8 (3)	C7—C2—C3—C4	1.2 (5)
O5^i^—Ni1—O1—C1	−169.2 (3)	C1—C2—C3—C4	−179.4 (3)
N1—Ni1—O1—C1	−82.9 (3)	C13—C12—C11—C10	0.4 (5)
N1^i^—Ni1—O1—C1	97.1 (3)	C14—C12—C11—C10	−171.1 (3)
O1—Ni1—N1—C9	146.8 (3)	C15—N2—C14—O4	178.1 (4)
O1^i^—Ni1—N1—C9	−33.2 (3)	C17—N2—C14—O4	−3.2 (6)
O5—Ni1—N1—C9	54.0 (3)	C15—N2—C14—C12	−4.0 (6)
O5^i^—Ni1—N1—C9	−126.0 (3)	C17—N2—C14—C12	174.7 (3)
O1—Ni1—N1—C13	−31.7 (2)	C13—C12—C14—O4	−57.3 (5)
O1^i^—Ni1—N1—C13	148.3 (2)	C11—C12—C14—O4	114.3 (4)
O5—Ni1—N1—C13	−124.5 (3)	C13—C12—C14—N2	124.7 (4)
O5^i^—Ni1—N1—C13	55.5 (3)	C11—C12—C14—N2	−63.7 (5)
Ni1—O1—C1—O2	−26.2 (5)	C2—C3—C4—C5	0.1 (6)
Ni1—O1—C1—C2	152.2 (2)	C2—C7—C6—C5	0.7 (5)
C9—N1—C13—C12	−2.3 (5)	C12—C11—C10—C9	−1.6 (5)
Ni1—N1—C13—C12	176.3 (2)	N1—C9—C10—C11	0.8 (6)
C11—C12—C13—N1	1.6 (5)	C3—C4—C5—C6	−1.0 (6)
C14—C12—C13—N1	173.7 (3)	C3—C4—C5—C8	179.0 (4)
C13—N1—C9—C10	1.1 (5)	C7—C6—C5—C4	0.7 (6)
Ni1—N1—C9—C10	−177.5 (3)	C7—C6—C5—C8	−179.4 (4)
C6—C7—C2—C3	−1.6 (5)	C14—N2—C17—C18	78.7 (5)
C6—C7—C2—C1	179.0 (3)	C15—N2—C17—C18	−102.4 (5)
O2—C1—C2—C3	3.7 (5)	C14—N2—C15—C16	−110.3 (5)
O1—C1—C2—C3	−174.9 (3)	C17—N2—C15—C16	71.0 (6)
O2—C1—C2—C7	−177.0 (3)	C4—C5—C8—O3	−175.0 (5)
O1—C1—C2—C7	4.5 (5)	C6—C5—C8—O3	5.1 (7)

Symmetry codes: (i) −*x*, −*y*, −*z*.

### Hydrogen-bond geometry (Å, °)

**Table:**

*D*—H···*A*	*D*—H	H···*A*	*D*···*A*	*D*—H···*A*
O5—H51···O4^ii^	0.84 (2)	1.97 (2)	2.796 (4)	170 (3)
O5—H52···O2	0.85 (3)	1.81 (3)	2.646 (4)	168 (4)

Symmetry codes: (ii) *x*−1, *y*, *z*.
